# Toward Producing Biopolyethylene/Babassu Fiber Biocomposites with Improved Mechanical and Thermomechanical Properties

**DOI:** 10.3390/polym16030419

**Published:** 2024-02-02

**Authors:** Eduardo da Silva Barbosa Ferreira, Fabiano Santana da Silva, Carlos Bruno Barreto Luna, Anna Raffaela de Matos Costa, Fernanda Menezes de Sousa, Laura Hecker de Carvalho, Renate Maria Ramos Wellen, Edcleide Maria Araújo

**Affiliations:** 1 Department of Materials Engineering, Federal University of Campina Grande, Campina Grande 58429-900, PB, Brazil; fabiano.santana@estudante.ufcg.edu.br (F.S.d.S.); brunobarretodemaufcg@hotmail.com (C.B.B.L.); fernandameneezes@outlook.com (F.M.d.S.); laura.hecker@professor.ufcg.edu.br (L.H.d.C.); edcleide.araujo@ufcg.edu.br (E.M.A.); 2 CICECO-Aveiro Institute of Materials, University of Aveiro, 3810-193 Aveiro, Portugal; anna.raffaela@ua.pt; 3 Department of Materials Engineering, Federal University of Paraíba, João Pessoa 58051-900, PB, Brazil; wellen.renate@propesq.ufpb.br

**Keywords:** biocomposites, biopolyethylene, babassu fiber, compatibilizing agents, properties

## Abstract

The development of polymeric biocomposites containing natural fibers has grown over the years due to the properties achieved and its eco-friendly nature. Thus, biocomposites involving a polymer from a renewable source (Biopolyethylene (BioPE)) and babassu fibers (BFs), compatibilized with polyethylene grafted with maleic anhydride (MA) and acrylic acid (AA) (PE-g-MA and PE-g-AA, respectively) were obtained using melt mixing and injection molded into tensile, impact, and HDT specimens. Babassu fiber was characterized with Fourier transform infrared spectroscopy (FTIR), thermogravimetry (TGA), and scanning electron microscopy (SEM). The biocomposites were characterized using torque rheometry, TGA, tensile strength, impact strength, thermomechanical properties, Shore D hardness, and SEM. The data indicate that the torque during the processing of compatibilized biocomposites was higher than that of BioPE/BF biocomposites, which was taken as an indication of a possible reaction between the functional groups. Compatibilization led to a substantial improvement in the elastic modulus, tensile strength, HDT, and VST and a decrease in Shore D hardness. These results were justified with SEM micrographs, which showed babassu fibers better adhered to the surface of the biopolyethylene matrix, as well as an encapsulation of these fibers. The system investigated is environmentally sustainable, and the results are promising for the technology of polymeric composites.

## 1. Introduction

The production of environmentally friendly materials as an alternative to replacing conventional polymer matrices and synthetic fibers has been increasingly sought by researchers and industry specializing in polymeric materials aiming at reduced environmental impacts caused by the use of petroleum-derived materials [1,2]. Natural fibers are considered a viable alternative for replacing some synthetic fibers to obtain polymeric composites due to their low density, biodegradability, abundance, and low cost, leading to materials with a good set of mechanical and thermomechanical properties [3,4]. Among the natural fibers most used in recent years by researchers and the specialized industry of plastic materials to obtain polymer composites are wood, jute, sisal, linen, and cotton, among others, which are considered an excellent alternative compared with the most common synthetic fibers [5]. The babassu fruit (*Orbignya speciosa)* is a very abundant fruit in the northeast region of Brazil, and the use of its fibers has been studied in recent years for the production of polymeric composites [6,7,8]. The primary use of babassu is its oil, which is used in industries ranging from medicine to food preparation. For oil production, the external part of the fruit (epicarp) is discarded, and thus, reusing the epicarp fibers as filler/reinforcement in the production of sustainable, completely bio-based, and biodegradable composites and biocomposites is highly desirable [9,10].

In addition to natural fibers, the use of bio-based polymer matrices, whether biodegradable or not, has been investigated to obtain commercially viable, entirely bio-based (biocomposites) with improved properties. These materials bring a very high ecological degree during their production process and are applied in various applications, such as aerospace, domestic, and automotive, among others [11,12,13,14,15,16,17,18]. Biopolymers such as poly (lactic acid) PLA, Polyhydroxybutyrate (PHB), and Polyhydroxyalkanoates (PHA) have been gaining prominence, and they are widely used in the manufacture of biocomposites [19]. Kannan et al. [20] studied the production of biocomposites involving PLA with sisal and jute fiber molded by pressing. It was observed that the composites with sisal fiber presented superior tensile and flexural strength compared with the composites with jute fiber, with better structural integrity, as an alternative for applications requiring superior damping properties. Within this class of polymers, biopolyethylene (BioPE) is a polymer with the same mechanical, chemical, and physical properties as conventional polyethylene but with an ethylene monomer obtained from sugarcane ethanol. In addition, during its production cycle, there is absorption of CO_2_, maintaining the environmental balance [21,22,23].

However, fiber/matrix adhesion in biocomposites involving non-polar polymers such as polyethylene and natural fibers, such as that from the babassu fruit, is poor due to the incompatible nature of the polymer and the natural fiber. For this kind of system, in order to obtain better fiber/matrix adhesion and mechanical properties, surface treatments need to be performed or compatibilizing agents need to be added to the formulations [24,25,26,27]. Xanthopoulou et al. [28] produced biocomposites based on recycled polyethylene with hemp fiber, compatibilized with PE-g-MA and a multifunctional polymeric chain extender. A high fiber content, up to 75%, was used. It was generally observed that compatibilizers promote adhesion in the system and, consequently, improve properties. Zhao et al. [29] studied the effect of sisal fiber content and compatibilization, using PE-g-MA, in polyethylene/sisal fiber composites, and found improvements in the mechanical properties of the studied materials.

These studies show that the use of compatibilizing agents with reactive groups in their structure is an excellent route to improve adhesion in the production of polymeric composites involving natural fibers and non-polar matrices. It is believed that the characteristic chemical groups in the compatibilizing agents react with the groups present in the babassu fiber, decreasing its polarity.

Thus, the current work aims to produce polymeric biocomposites of biopolyethylene/babassu fiber using melt mixing and to study the influence of compatibilization of the biocomposites using polyethylene grafted with MA and AA. The development of compatibilized biocomposites using biopolyethylene and babassu fibers is a novelty in the literature. The biocomposites produced here were evaluated based on their rheological (torque rheometry), thermal (Thermogravimetry), mechanical (tensile, impact, Shore D hardness), thermomechanical (HDT and VST), and morphological (SEM) properties.

## 2. Methodology

### 2.1. Materials

Braskem’s SHC7260 (São Paulo, Brazil), a high-density polyethylene with a density of 0.959 g/cm^3^ and an MFI of 7.2 g/10 min (190 °C/2.16 kg) was used as the matrix and henceforth called “BioPE”. BioPE is a 100% recycled biopolymer made from ethanol obtained from sugar cane. The substitution of petroleum-based polyethylene with Bio-PE is important for reducing CO_2_ in the atmosphere. Babassu fiber, scientifically known as *Orbignya speciosa*, was labeled BF. Addivant (Danbury, CT, USA) supplied Polybond 3029 (PE-g-MA), with 1.5–1.7% MA, a density of 0.95 g/cm^3^, MFI of 4 g/10 min (190 °C/2.16 kg), and Polybond 1009 (PE-g-AA), with 5.5–6.5% AA, density of 0.95 g/cm^3^, and an MFI of 5 g/10 min, were used as compatibilizers.

### 2.2. Methods

Prior to use, babassu fibers were washed with distilled water to remove any impurities from their surface. BF (80 °C), BioPE, PE-g-MA, and PE-g-AA (60 °C) were dried for 24 h before processing. Then, the materials were melt mixed in a Thermo Fisher Scientific internal mixer (Waltham, MA, USA), model Haake Rheomix 3000, at 170 °C and 60 rpm for 10 min. The rotor used in the internal mixer is the roller type, which is suitable for mixing materials in the molten state. Matrix and filler (70/30) were manually mixed and added to the mixing chamber of the rheometer. The compatibilizers (10 and 20%) were added after 2 min of processing. The compositions investigated as well as neat BioPE, processed under the same conditions described above, are depicted in Table 1.

**Figure 1:**
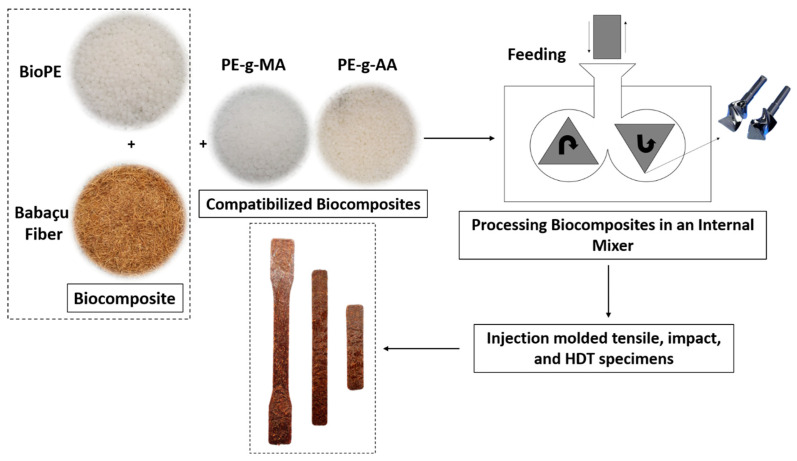
Production scheme of the BioPE/BF biocomposites and the compatibilized biocomposites.

After melt blending, the biocomposites were ground in a Laboremus (Paraíba, Brazil) TP 150 knife mill. The ground biocomposites were vacuum-dried for 24 h at 60 °C prior to being injection molded using an Arburg Allrounder 207C Golden Edition machine (Radevormwald, Germany) to produce tensile (ASTM D638-Type I [30]), impact (ASTM D256 [31]), and HDT (ASTM D648 [32]) specimens. The injection molding apparatus temperature profile was 170/170/175/175/180 °C with a mold temperature of 20 °C. Figure 1 illustrates the processing scheme of the BioPE/BF biocomposites and the compatibilized biocomposites.

### 2.3. Characterizations

Fourier transform infrared spectroscopy (FTIR) analysis was conducted on babassu fiber utilizing a BRUKER (Billerica, MA, USA) Alpha II spectrometer with an ATR module. The scanning was 4000–400 cm^−1^ with a 4 cm^−1^ resolution to detect the main functional groups.

Thermogravimetric (TGA) analysis was performed on babassu fiber, BioPE, and the biocomposites using a Shimadzu DTG 60H (Kyoto, Japan). Heating was performed on 5 mg samples in a nitrogen atmosphere at a rate of 10 °C/min from room temperature to 600 °C with a gas flow rate of 50 mL·min^−1^.

VEGA 4 - TESCAN (Brno, Czech Republic) equipment was used to perform scanning electron microscopy analysis of babassu fiber and the biocomposites at 30 kV. For the biocomposites, the gold-sputtered fracture surfaces of the impact specimens were analyzed.

Rheometric curves were obtained by performing torque rheometry in a Thermo Fisher Scientific Haake Rheomix 3000 mixer (Waltham, MA, USA) operating with roller-type rotors for 10 min at 170 °C and 60 rpm.

The ASTM D 638 [30] standard was used to perform tensile tests on specimens to determine their elastic modulus and tensile strength. The experiment was carried out at room temperature conditions using an Oswaldo Filizola BME machine (São Paulo, Brazil) with a 10 kN load cell and 5 mm·min^−1^ deformation rate.

Izod impact strength was determined on Ceast Resil 5.5 (Turin, Italy) equipment. The experiment was carried out at room temperature on notched specimens and a 2.75 J pendulum following the ASTM D 256 [31] standard. The reported results are average values of 10 samples per composition.

Ceast equipment HDT 6 VICAT/N 6921.000 (Turin, Italy) was used to measure thermomechanical properties. HDT (ASTM D 648 [32]) and VST (ASTM D1525 [33]) were measured under a load of 455 kPa for HDT and 50N for VST with 120 °C·h^−1^. The analysis was based on an average of 3 specimens.

The ASTM D2240 [34] standard was used to determine resistance to penetration in Shore-Durometer Hardness Type “D” equipment, MetroTokyo (São Paulo, Brazil), 50N load. The reported results are an average of 5 samples.

## 3. Results and Discussion

### 3.1. Characterization of Babassu FIBER

#### 3.1.1. Fourier Transform Infrared Spectroscopy (FTIR)

FTIR, thermogravimetry, and SEM were performed to analyze the characteristics of babassu fiber.

**Figure 2:**
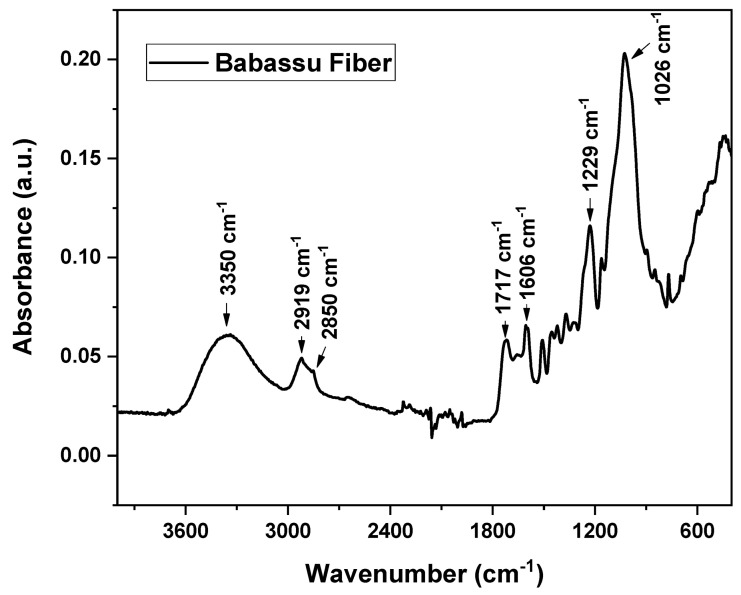
FTIR spectra of babassu fiber.

The babassu fiber FTIR spectrum is shown in Figure 2. At 3350 cm^−1^, there is a broadband attributed to the stretching of the hydroxyl groups of the fiber, as well as associated with adsorbed water, which is very common in natural fibers [35,36]. At 2919 and 2850 cm^−1^, there are peaks attributed to the C-H stretching of the alkyl and methylene groups present in cellulose, hemicellulose, and lignin of babassu fiber [37]. The bands at 1717 and 1606 cm^−1^, are attributed to C=O (esters) and functional groups such as ketone and carboxylic acids, which are derived from hemicellulose and lignin. The bands at 1229 and 1026 cm^−1^ are characteristic of the C-O stretching of cellulose [35,38,39].

#### 3.1.2. Thermogravimetry (TGA)

**Figure 3:**
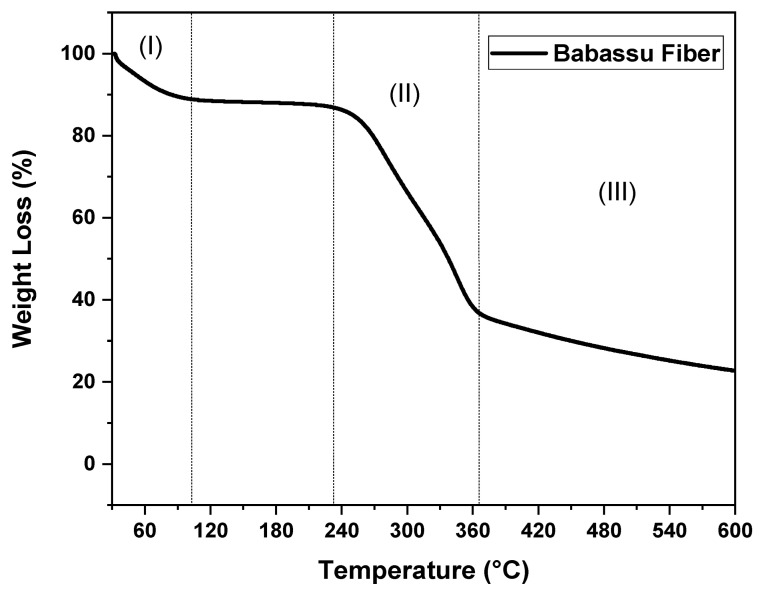
TGA curve of babassu fiber.

Thermogravimetric analysis was performed to observe the thermal stability of the babassu fiber (BF), and its curve is shown in Figure 3. Babassu fiber has three stages of decomposition, typical of natural fibers. The first stage, characteristic of water loss, i.e., moisture evaporation, is around 32–100 °C, with a mass loss of approximately 13%. The decomposition temperature of 10% mass (T_D10%_) is around 83.3 °C [40]. The second stage of decomposition is characteristic of hemicellulose and cellulose, around 225–370 °C, with a mass loss of 50% [41]. The 50% weight loss temperature observed in Figure 3 was 337.5 °C. The third and last stage is the final decomposition of hemicellulose and cellulose and the beginning of lignin decomposition, starting at 370 °C, with a slower decomposition rate but with a mass loss of approximately 15% [42]. It can be noted that the babassu fiber presents a residue at 600 °C of 22.6% by mass, which is characteristic of carbonaceous material formed after the decomposition of hemicellulose, cellulose, and lignin.

#### 3.1.3. Scanning Electron Microscopy (SEM)

**Figure 4:**
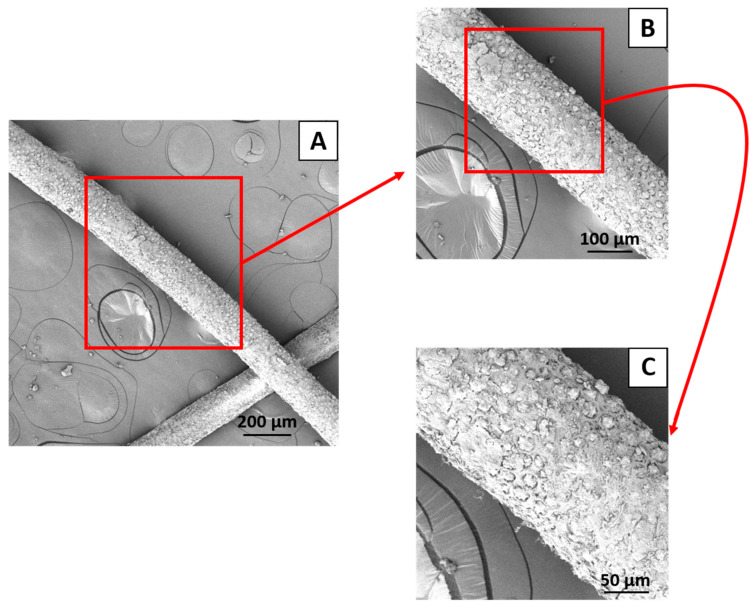
SEM images of babassu fiber at 200× (**A**), 500× (**B**), and 1000× (**C**).

SEM images of the babassu fiber, with magnifications of 200, 500, and 1000×, respectively, are shown in Figure 4A–C. The babassu fiber has a long format and a diameter of approximately 170 µm, estimated using Image J software, version 1.51k. Figure 4C shows that the surface of babassu fiber is porous and rough, which is beneficial for improving interfacial adhesion, mainly when using suitable compatibilizers. Babassu fiber pores have an average size of around 12 µm. Pilla et al. [43] observed that the porosity of wood fibers positively influences the properties of the material when using an ideal compatibilizer.

### 3.2. Characterization of Biocomposites

#### 3.2.1. Torque Rheometry

**Figure 5:**
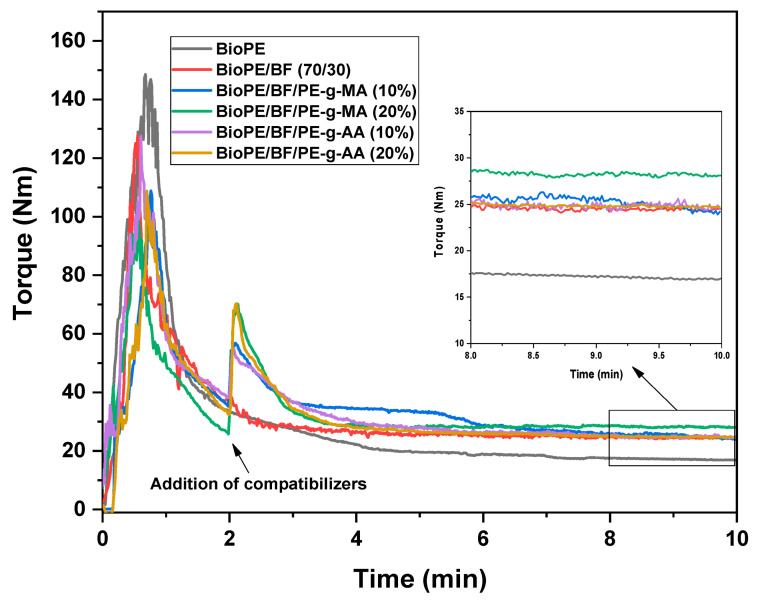
Rheometric curves of BioPE, BioPE/BF, and the compatibilized biocomposites.

The rheometric curves for BioPE, the BioPE/BF biocomposites, and the compatibilized biocomposites with 10 and 20% compatibilizers are depicted in Figure 5. Using torque rheometry, it is possible to detect degradation, chemical interactions, and processing improvement [44,45,46].

Figure 5 shows a sudden torque increase in the first minute for all systems, characteristic of material feeding in the rheometer. As the material melts, torque decreases and then tends to stabilize. By adding 30% babassu fiber, the final torque increases, which indicates an increase in the viscosity. BF restricts the mobility of chains and decreases matrix fluidity. In the specialized literature on polymers, it is noted that adding a natural filler to a polymeric matrix hinders the flow of the molten material, increasing the torque and thus the viscosity of the materials [47].

After 2 min of processing, 10 and 20% of the compatibilizers were added to the BioPE/BF composition to obtain the compatibilized biocomposites. The 8–10 min average torque values were 17.22, 24.51, 25.26, 28.1, 24.85, and 24.82 for BioPE, BioPE/BF, BioPE/BF/PE-g-MA (10%), BioPE/BF/PE-g-MA (20%), BioPE/BF/PE-g-AA (10%), and BioPE/BF/PE-g-AA (20%), respectively. The addition of compatibilizers increased the average torque compared with the BioPE/BF biocomposites. This behavior is thought to indicate improved interactions between BioPE and babassu fiber, possibly through reaction chemistry, with maleic anhydride (MA) and acrylic acid (AA) reacting with the hydroxyls of cellulose present in babassu fiber, leading to esterification reactions and hydrogen bonds [37,48,49]. The torque curves also indicate that all systems investigated did not significantly degrade during processing as a plateau was reached after melting.

It is worth noting that the biocomposites containing PE-g-MA, mainly the one with 20%, were those that presented the highest torque among the studied systems, which is considered the most efficient compatibilizer. The addition of 10 and 20% PE-g-AA did not significantly change the torque of the BioPE/BF composite. As observed for the mechanical properties presented next, mainly the tensile strength results confirm PE-g-MA to be the most effective compatibilizer, particularly at the highest concentration (20%) investigated.

#### 3.2.2. Thermogravimetry (TGA)

**Figure 6:**
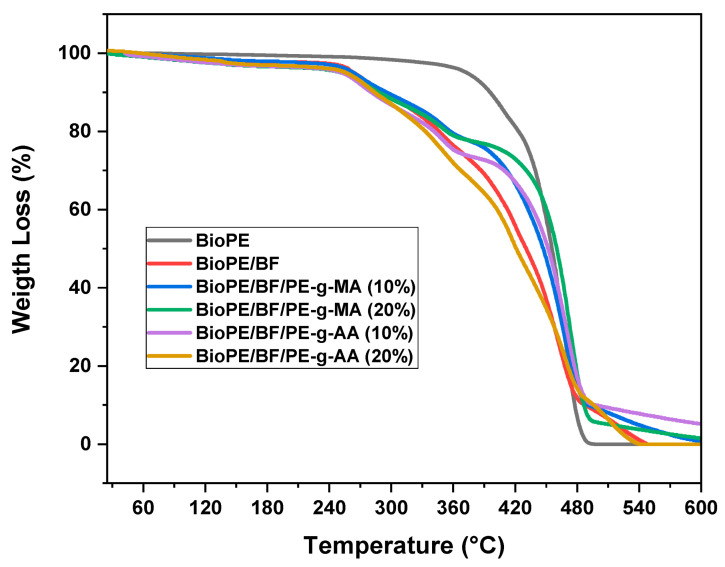
TGA curves of BioPE, BioPE/BF, and the compatibilized biocomposites.

The TGA curves of BioPE, BioPE/BF, and the compatibilized biocomposites are displayed in Figure 6. BioPE has good thermal stability, with decomposition starting around 330 °C and ending around 490 °C. This indicates that for the processing temperature used in the production of materials, no significant thermal degradation should occur.

Figure 3 shows that the thermal stability of the babassu fiber biocomposites was lower than that of the matrix, which was associated with the characteristic low thermal stability of natural fibers [36,50]. For the biocomposites, it is possible to observe the presence of three stages of decomposition: the first is characteristic of the water loss characteristic of babassu fiber, up to approximately 250 °C; then, the second mass loss, reaching around 385 °C, is characteristic of the decomposition of babassu fiber components (cellulose, hemicellulose, and lignin), as mentioned before; and the third and last stage of decomposition is characteristic of the decomposition of BioPE, as well as of the residual lignin present in the fibers.

Table 2 shows the decomposition parameters of BioPE and the studied biocomposites. It shows that the T_D10%_ (the temperature at which there is 10% mass loss) of BioPE is the highest (395 °C) because it presents the highest initial thermal stability, as mentioned previously. With the addition of babassu fiber, there is a decrease in this temperature because the fiber has low thermal stability and there is a low interaction between BioPE and natural fiber, reaching values of 280–295 °C. It is observed that T_D50%_ (the temperature at which there is 50% mass loss) decreased by 25 °C with the addition of 30% babassu fiber to BioPE. However, with the addition of the compatibilizers, T_D50%_ increases by up to 30 °C, reaching up to 460 °C for BioPE/BF/PE-g-MA (20%). This is due to the improved compatibility, which considerably improves the interfacial adhesion and possible interactions between the phases studied, as observed in the results of torque rheometry, tensile properties, and SEM images.

According to Poletto [51], with the improvement in surface adhesion, as well as the interactions between composite phases with the addition of a compatibilizing agent, there is an improvement in the thermal stability of the systems compared with the biocomposites without a compatibilizing agent.

#### 3.2.3. Tensile Test

**Figure 7:**
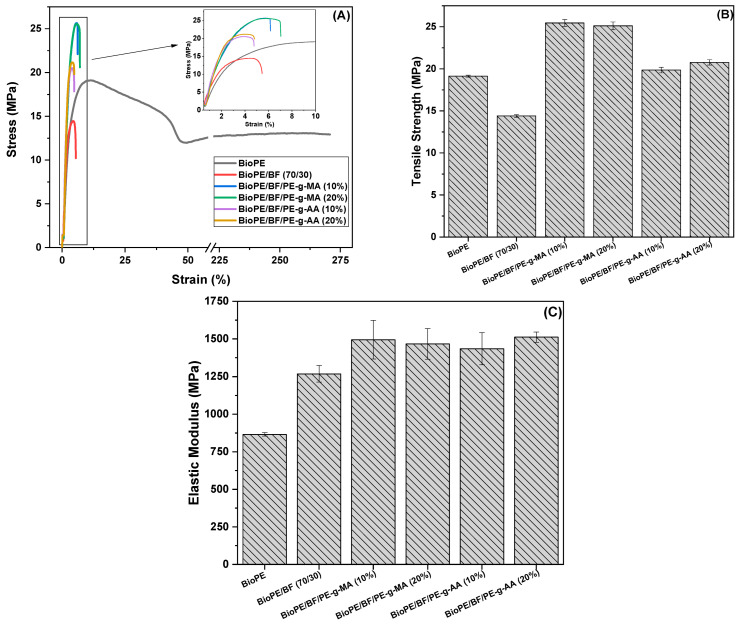
Stress–strain curves (**A**), tensile strength results (**B**), and elastic modulus (**C**) of the systems studied.

Tensile stress–strain curves, tensile strength, and the elastic modulus for BioPE, BioPE/BF, and the compatibilized biocomposites are displayed in Figure 7.

From the stress–strain curves observed in Figure 7A, it is possible to observe that BioPE presents a high elongation at break, reaching approximately 270%. The elongation at break decreases (4.9%) for the BioPE/BF biocomposites. However, adding PE-g-MA compatibilizer increases the elongation at break, reaching up to 7% for BioPE/BF/PE-g-MA (20%). PE-g-AA does not increase the elongation at break, despite increased biocomposite tensile strength. The greatest tensile strength obtained for the PE-g-MA compatibilized biocomposites is observed in the stress–strain curves (Figure 7A), which are shown as a zoomed-in view in Figure 7B.

Figure 7B shows that BioPE has a tensile strength of around 19.1 MPa, characteristic of its elasticity, as observed in the stress–strain curve, and does not require more significant stress for deformation to occur. The addition of 30% babassu fiber to BioPE led to a reduction in tensile strength and elongation. This is attributed to the poor adhesion between the polymer matrix (non-polar) and the babassu fiber (polar) [52]. This decrease in strength with the addition of natural fiber to a polymeric matrix was observed in the literature [11,14,53]. The addition of compatibilizers led to an increase in the tensile strength of BioPE/BF, surpassing even that of neat BioPE. The elongation at break was either unchanged or slightly decreased. These results are interpreted as being due to an improved polymer/fiber interface promoted by the reactive groups in the compatibilizers. It was observed that the PE-g-MA compatibilizer was more efficient compared with the PE-g-AA, with tensile strength reaching values of up to 25.5 MPa. This indicates that the maleic anhydride graft is more efficient than the acrylic acid graft and promotes a greater degree of interaction with the babassu fiber.

The elastic modulus results for the materials studied are depicted in Figure 7C. Neat BioPE presents a low elastic modulus, around 865.2 MPa. With the addition of BF, there is an increase in the stiffness of the material, reaching approximately 1268 MPa, due to the addition of the high content of a rigid filler, which hinders the free mobility of the BioPE chains. These data are compatible with those obtained with torque rheometry. The stiffness of the materials increased to 1434–1512 MPa when 10 and 20% of either one of the studied compatibilizers were added. Contrary to what was observed in torque rheometry, the elastic modulus values for the compatibilized systems were equivalent within experimental error, and no difference in the effectiveness of compatibility between the compatibilizers was observed. With adequate compatibility, as observed in the SEM images and torque rheometry, there was an increase in the elastic modulus for both compatibilizers, indicating good interactions in the studied systems.

#### 3.2.4. Impact Test

**Figure 8:**
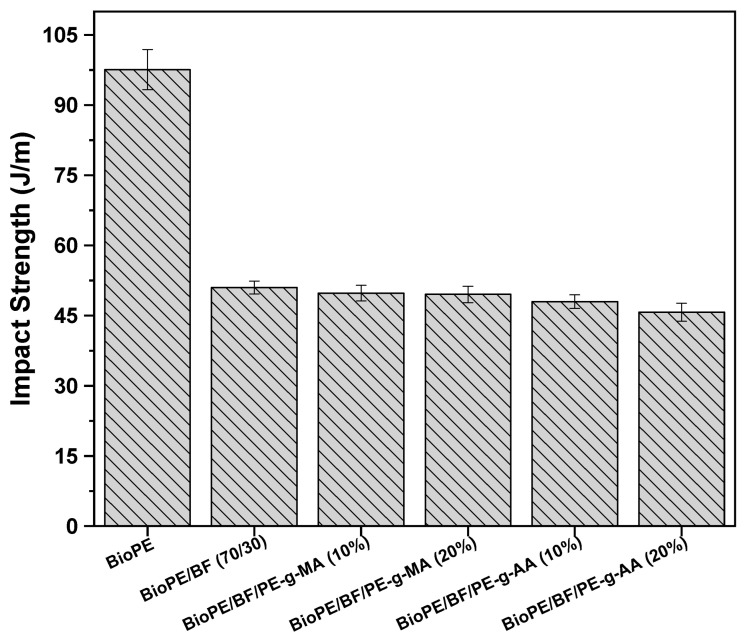
Impact strength of BioPE and the biocomposites.

The impact strength of BioPE, the BioPE/BF biocomposite, and the compatibilized biocomposites with 10 and 20% of the compatibilizers are presented in Figure 8. BioPE has 97.5 J/m of impact strength, characteristic of a ductile material, as observed in the stress–strain curve, and presents an elongation of approximately 260%. For BioPE/BF, this property value decreased, reaching approximately 51 J/m. This sudden decrease in the property was expected due to the high content of babassu fiber used, as well as the babassu fiber acting as stress a concentrator, forming agglomerates. This consequently reduces elongation at break and the amount of energy absorbed, thus facilitating the fracture of the material. In addition, babassu fiber is considered to be a rigid filler.

Taking into consideration the experimental error, no significant difference in impact strength, compared with the BioPE/BF biocomposite, was observed with the addition of 10 and 20% of either one of the compatibilizers. The elongation at break of all the biocomposites was similar, which explains the result obtained.

Although the impact strength declines as babassu fiber is added, the value of this property is similar to those of the polymers widely used in the plastics industry today, such as polypropylene, polystyrene, and poly (lactic acid).

#### 3.2.5. Heat Deflection Temperature (HDT)

**Figure 9:**
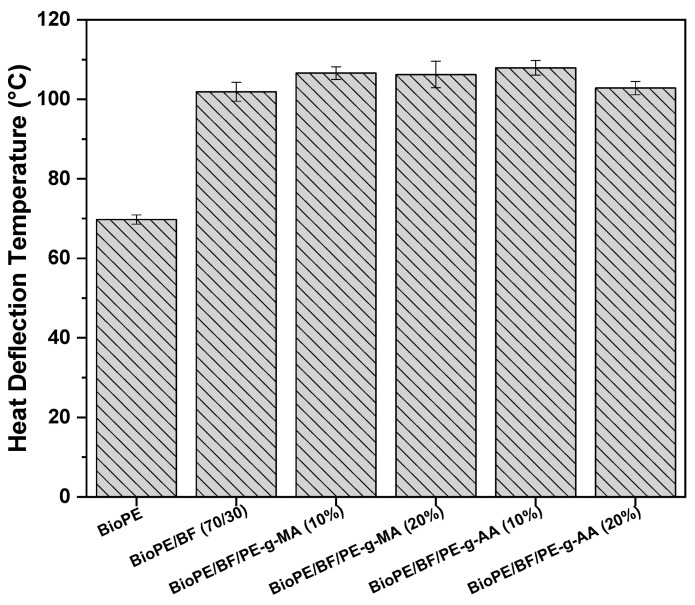
HDT of BioPE, the BioPE/BF biocomposites, and the compatibilized biocomposites.

HDT is one of the most sought-after parameters by researchers, industry, and product designers, as it is directly related to a material’s ability to be used for a given application. The test consists of the temperature at which the tested material deflects 0.25 mm when subjected to mechanical stress and controlled heating [48,54,55]. Figure 9 shows the HDT results of BioPE, the BioPE/BF biocomposites, and the biocomposites compatible with PE-g-MA and PE-g-AA.

Our data shows that the HDT of BioPE is approximately 69 °C, which agrees with the data presented in the literature [44]. With the addition of 30% babassu fiber, this property significantly increased, reaching values of approximately 101.9 °C, which is 46.1% higher than that of neat BioPE. This increase is associated with the increase in stiffness of the material when 30% of the natural fiber was added, as observed in the elastic modulus results (Figure 7C). This means that greater energy (temperature) is required for the material to deflect, and, therefore, the dimensional stability for the biocomposites is improved when compared with that of the neat matrix [56]. According to Akil et al. [57], HDT values increase with fiber content and the length of the natural fiber. Increasing the natural fiber content reduces the mobility of polymeric chain segments and increases thermomechanical stability.

By adding 10% and 20% of the compatibilizers, the HDT was further increased to around 107.9 °C. This improved dimensional stability of the biocomposite is attributed to better interaction between the reactive groups of the compatibilizer and BF, ultimately reducing the interfacial tension between the matrix and natural fiber. Meekum [58] observed that the graphting of maleic anhydride in PE/PET blends to obtain composites with rice husk fiber improves the interfacial adhesion of the system, with a positive effect on the efficiency of the reinforcing natural fiber and an improvement in the HDT of the studied systems.

#### 3.2.6. Vicat Softening Temperature (VST)

**Figure 10:**
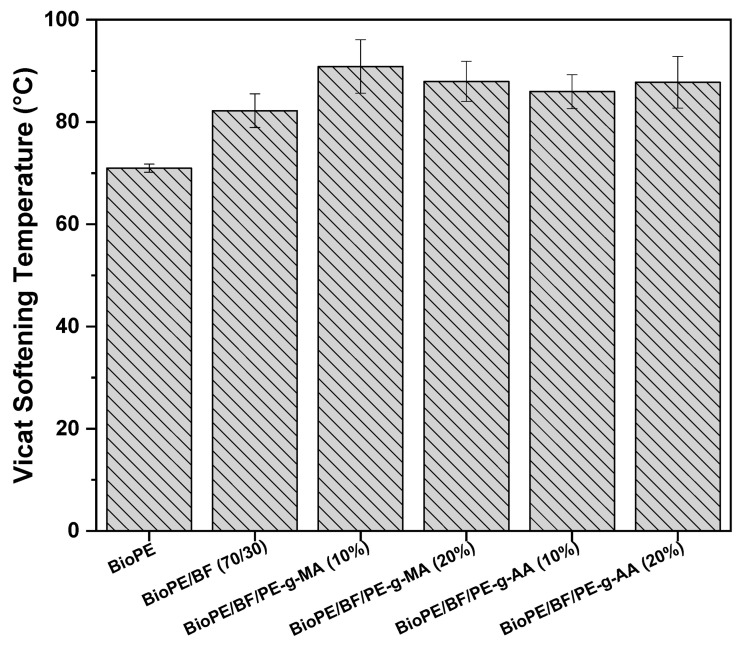
VST of BioPE, the BioPE/BF biocomposites, and the compatibilized biocomposites.

Another thermomechanical property that is widely explored in polymeric biocomposites and essential for the design of industrial applications is the Vicat softening temperature (VST). The VST is considered as the temperature where there is a penetration of 1 mm by a needle subjected to a specific load and temperature under controlled heating [59]. Figure 10 shows the results obtained from the Vicat softening temperature (VST) test for BioPE, the BioPE/BF biocomposites, and the compatibilized biocomposites.

**Figure 11:**
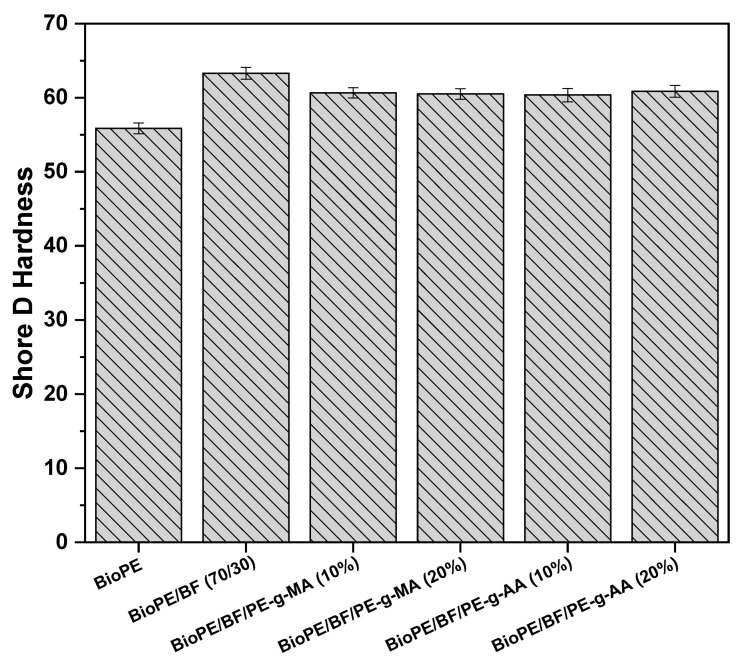
Shore D hardness of BioPE, the BioPE/BF biocomposites, and the compatibilized biocomposites.

BioPE has a Vicat softening temperature of approximately 71 °C. With the addition of babassu fiber, there is an increase of approximately 11 °C, reaching a value of 82 °C. This expressive increase, similar to what was observed for HDT, is due to the addition of a rigid load to the thermoplastic, causing an increase in the material’s rigidity, as observed in the results of elastic modulus (Figure 7C). It is also due to the greater hardness provided with the addition of 30% babassu fiber, as will be later observed in the Shore D hardness results (Figure 11). The compatibilizers further increased the Vicat softening temperature up to 90.8 °C, with 10% PE-g-MA. This increase is associated with the formation of a more homogeneous interface and an improvement in the interactions between the phases.

Analyzing the results of HDT and VST, the significant contribution of babassu fiber addition is evident, as well as that of both compatibilizing agents, which led to a substantial improvement in the thermomechanical stability of the systems investigated. These results are critical from a scientific and technological perspective.

#### 3.2.7. Shore D Hardness

The hardness results of all systems investigated are displayed in Figure 11. BioPE outperforms others in flexibility with the lowest surface hardness of around 55 Shore D, as shown in Figure 7C and Figure 8. The addition of 30% babassu fiber increases the surface hardness by 14% compared with BioPE, reaching values of approximately 63 Shore D. This is attributed to the addition of a rigid filler, restricting the movement of polymer chains. Consequently, the biocomposites show greater resistance to penetration. Satapathy and Kothapalli [60] observed that the addition of 10% banana fiber is not enough to increase the Shore D hardness of a pure polyethylene residue, which requires the addition of 30% natural fiber to show significant increases in the property compared with the studied matrix.

With the addition of 10 and 20% of the compatibilizers, there was a slight reduction in the surface hardness of the materials, reaching close to 61 Shore D. But the results are still higher than that of BioPE. The Shore D hardness values obtained for the compatibilized biocomposites are close to the BioPE/BF result, considering experimental error.

The results obtained for Shore D hardness, HDT, VST, elastic modulus, and tensile strength, indicate that babassu fiber effectively reinforces BioPE and that the addition of the studied compatibilizers further improves these properties, producing technologically important biocomposites.

#### 3.2.8. Scanning Electron Microscopy (SEM)

**Figure 12:**
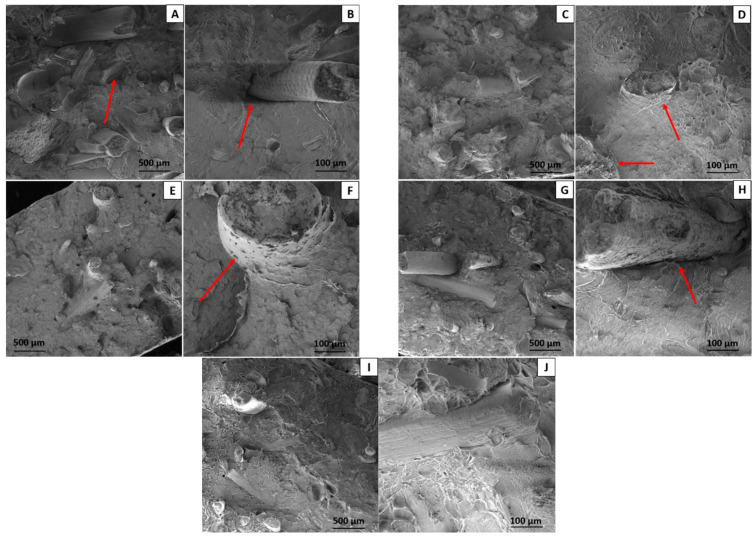
Fracture surface obtained with SEM of BioPE/BF (**A**,**B**), BioPE/BF/PE-g-MA (10%) (**C**,**D**), BioPE/BF/PE-g-MA (20%) (**E**,**F**), BioPE/BF/PE-g-AA (10%) (**G**,**H**), and BioPE/BF/PE-g-AA (20%) (**I**,**J**) at 100× and 500× magnification.

SEM images of BioPE/BF biocomposites and biocomposites compatibilized with 10% and 20% of both compatibilizers at 100× and 500× magnification are displayed in Figure 12.

Figure 12A,B shows the biocomposites with 30% babassu fiber. As seen in the specialized literature [24], natural fibers present a low adhesion with conventional thermoplastics due to their polar nature. Thus, it is possible to notice a high number of voids on the surface, as indicated by the arrow in Figure 12A. Fiber pull-out and fiber agglomerates were also observed on the surface, indicating poor filler dispersion and low fiber/matrix adhesion. Figure 12B shows that the fiber presents a well-defined interface concerning the biopolyethylene matrix, which is characteristic of the low adhesion mentioned among the present phases.

After adding 10% and 20% PE-g-MA to the biocomposites, there was a noticeable improvement in compatibility, resulting in fewer voids and agglomerates (see Figure 12C–F) as well as significantly improved fiber/matrix adhesion. Fiber encapsulation is observed in Figure 12D,F, as indicated by the arrows [48,61,62]. This improves adhesion among the matrix and BF, with the BF becoming more attached to the BioPE surface due to interactions among OH present in the babassu fiber and the maleic anhydride. The improved adhesion with the addition of PE-g-MA led to fiber fracture in the plane (Figure 12D), indicating a transfer of tension between BioPE and the babassu fiber.

Regarding 10 and 20% PE-g-AA (Figure 12G–J), it is also noticed that the number of agglomerates and voids on the fracture surface decreased and that with 10% PE-g-AA, the morphology was not as good as that of the system with 10% PE-g-MA. The system compatibilized with 10% PE-g-AA shows relatively poor fiber/matrix adhesion, as observed in Figure 12H. The addition of 20% PE-g-AA, led to an increase in interactions between BF and BioPE, and thus to a system with greater morphological homogeneity.

Thus, the addition of the compatibilizers investigated here led to an improvement in tensile strength, HDT, and VST properties, particularly for the biocomposites compatibilized with PE-g-MA.

## 4. Conclusions

Biocomposites using a biopolymer from a renewable source (BioPE) and a natural fiber abundant in the Brazilian northeast region (babassu fiber), were obtained using melt mixing and molded with injection. The BioPE/BF biocomposites without compatibilizing agents showed low adhesion between the components, as observed with SEM, acquiring lower mechanical properties but significantly improved thermal and thermomechanical stability compared with BioPE. With the addition of compatibilizing agents, the average torque increased compared with the unmodified biocomposites. This suggests that a reaction occurred between the components, resulting in improved adhesion between the BF and BioPE. This was confirmed by improvements in properties like the elastic modulus, tensile strength, HDT, and VST. There was an increase in T_D50%_ for TGA. Although Biopolyethylene is not biodegradable, its use and the addition of babassu fiber help to minimize the development of composites from non-renewable sources, thus generating a more sustainable aspect. Furthermore, as it is a very abundant fiber in the northeast region of Brazil, it can generate environmental and economic benefits associated with cultivating these fibers, generating resources for the communities that cultivate them and reducing the final cost of biocomposites. Thus, our results confirm that babassu fiber can be used as reinforcement for biopolyethylene. The specialized polymer industry can benefit from the addition of up to 20% PE-g-MA or PE-g-AA to enhance properties.

## Figures and Tables

**Table 1:** Compositions of BioPE/babassu fiber biocomposites.

Samples	BioPE (%)	Babassu Fiber (%)	PE-g-MA (%)	PE-g-AA (%)
BioPE	100	-	-	-
BioPE/BF	70	30	-	-
BioPE/BF/PE-g-MA	60	30	10	-
BioPE/BF/PE-g-MA	50	30	20	-
BioPE/BF/PE-g-AA	60	30	-	10
BioPE/BF/PE-g-AA	50	30	-	20

**Table 2:** Parameters T_D10%_, T_D50%_, and T_D99.9%_ of BioPE and the biocomposites.

Samples	T_D10%_ (°C)	T_D50%_ (°C)	Residue at 600 °C (%)
BioPE	395	455	0.0
BioPE/BF	292	430	0.0
BioPE/BF/PE-g-MA (10%)	295	447	0.6
BioPE/BF/PE-g-MA (20%)	287	460	1.4
BioPE/BF/PE-g-AA (10%)	280	451	5.0
BioPE/BF/PE-g-AA (20%)	283	430	0.0

## Data Availability

Data are contained within the article.
